# Predicting Efficacies of Fractional Doses of Vaccines by Using Neutralizing Antibody Levels: Systematic Review and Meta-Analysis

**DOI:** 10.2196/49812

**Published:** 2024-07-12

**Authors:** Zhanwei Du, Caifen Liu, Yuan Bai, Lin Wang, Wey Wen Lim, Eric H Y Lau, Benjamin J Cowling

**Affiliations:** 1 WHO Collaborating Centre for Infectious Disease Epidemiology and Control, School of Public Health, Li Ka Shing Faculty of Medicine, The University of Hong Kong, Hong Kong, China (Hong Kong); 2 Laboratory of Data Discovery for Health Limited (D24H), Hong Kong Science and Technology Park, Hong Kong, China (Hong Kong); 3 Department of Genetics, University of Cambridge, Cambridge, United Kingdom

**Keywords:** COVID-19, SARS-CoV-2, dose fractionation, neutralizing antibody level, vaccination, review, vaccine

## Abstract

**Background:**

With the emergence of SARS-CoV-2 variants that have eluded immunity from vaccines and prior infections, vaccine shortages and vaccine effectiveness pose unprecedented challenges for governments in expanding booster vaccination programs. The fractionation of vaccine doses might be an effective strategy for helping society to face these challenges, as fractional doses may have efficacies comparable with those of the standard doses.

**Objective:**

This study aims to investigate the relationship between vaccine immunogenicity and protection and to project efficacies of fractional doses of vaccines for COVID-19 by using neutralizing antibody levels.

**Methods:**

In this study, we analyzed the relationship between in vitro neutralization levels and the observed efficacies against both asymptomatic infection and symptomatic infection, using data from 13 studies of 10 COVID-19 vaccines and from convalescent cohorts. We further projected efficacies for fractional doses, using neutralization as an intermediate variable, based on immunogenicity data from 51 studies included in our systematic review.

**Results:**

In comparisons with the convalescent level, vaccine efficacy against asymptomatic infection and symptomatic infection increased from 8.8% (95% CI 1.4%-16.1%) to 71.8% (95% CI 63%-80.7%) and from 33.6% (95% CI 23.6%-43.6%) to 98.6% (95% CI 97.6%-99.7%), respectively, as the mean neutralization level increased from 0.1 to 10 folds of the convalescent level. Additionally, mRNA vaccines provided the strongest protection, which decreased slowly for fractional dosing with dosages between 50% and 100% of the standard dose. We also observed that although vaccine efficacy increased with the mean neutralization level, the rate of this increase was slower for vaccine efficacy against asymptomatic infection than for vaccine efficacy against symptomatic infection.

**Conclusions:**

Our results are consistent with studies on immune protection from SARS-CoV-2 infection. Based on our study, we expect that fractional-dose vaccination could provide partial immunity against SARS-CoV-2 and its variants. Our findings provide a theoretical basis for the efficacy of fractional-dose vaccines, serving as reference evidence for implementing fractional dosing vaccine policies in areas facing vaccine shortages and thereby mitigating disease burden. Fractional-dose vaccination could be a viable vaccination strategy comparable to full-dose vaccination and deserves further exploration.

## Introduction

COVID-19 continues to threaten fragile health care and socioeconomic systems, exacting a devastating human and economic toll around the world. The primary means of COVID-19 control are the widespread implementation of vaccination and the preservation of public health and social measures. Worldwide, 123 vaccine candidates have been tested in humans as of August 31, 2022, with 52 in the final phases of clinical trials [1]. Although 68% of the world population has received at least one dose of the COVID-19 vaccine as of October 1, 2022 [2], global vaccine shortages and inequities persist [3], with only 18.55% and 1.27% of people in low-income countries being fully vaccinated or receiving booster doses, respectively [2].

As of October 2022, the Omicron subvariant BA.5 has displaced BA.2 as the predominant strain of SARS-CoV-2 in countries around the world. The timely achievement of administering the optimal threshold of doses to the population was critical to preventing a new mutation of the virus, thereby averting accelerated transmission dynamics in society and mitigating consequential socioeconomic issues [4-6]. Although vaccine effectiveness is expected to wane quickly, a booster shot can restore the protection against infection by both Omicron subvariants to 30%‐60% and increase protection against severe disease from a high level to a very high level [7]. A highly vaccinated population is still not enough to combat Omicron’s spread due to immune escape and the waning of vaccine-derived immunity against the ancestral SARS-CoV-2 strain [8]. Countries should time the ramp-up of their booster doses to account for the risks of waning vaccine effectiveness against infection, disease, and death. Mass vaccination with fractional doses of COVID-19 vaccines to boost immunity in a vaccinated population could be a cost-effective strategy for mitigating the public health costs of resurgences caused by vaccine-evasive variants [9].

Given accelerating vaccination, expanding booster programs, and concerns about vaccine safety, the fractional dosing of vaccines could help by providing partial protection for a significant number of people [10]. In the clinical trial of the mRNA-1273 vaccine, 2 fractional doses (half of the full dose) gave geometric mean plaque reduction neutralization test_80_ titers that, after 2 weeks, were comparable with those of 2 standard doses [11]. Further, the model-predicted efficacy of the half dose and the measured efficacy of the standard dose were nearly 95% for symptomatic disease [12]. Fractional-dose vaccination has also successfully addressed vaccine shortages in outbreak events [13]. For example, Angola and the Democratic Republic of the Congo adopted fractional doses (one-fifth of the standard dose) of the 17DD yellow fever vaccine to accelerate vaccine rollout during their 2016 yellow fever outbreaks and finally won the war against yellow fever [14,15]. During the mpox (monkeypox) outbreaks in 2022, the US Food and Drug Administration (FDA) issued an emergency use authorization on August 9, 2022, for the JYNNEOS vaccine to prevent mpox infections by administering intradermal injections with a lower volume (one-fifth) to adults aged ≥18 years [16]. During the COVID-19 pandemic period, fractional dosing for young people was approved in some countries, considering that young adults have a robust immune response to vaccines, and lower doses may also be linked with fewer side effects. For example, the FDA approved the use of one-third of the standard doses of the Pfizer-BioNTech COVID-19 vaccine (10 μg) for 5- to 11-year-old children, and one-tenth doses (3 μg) are used for children aged <5 years [17-19].

Fractional-dose vaccination could be an effective strategy for mitigating the epidemic risks of COVID-19 (eg, it allows for the deployment of more vaccines to reach more individuals in settings with limited health care budgets [12,20]) and reducing its disease burden, and this strategy’s effectiveness may be comparable to that of the full-dose strategy [21,22]. However, vaccine efficacy has mainly been tested for standard doses (ie, via clinical trials) [23] rather than for fractional doses, of which the efficacies are measured via phase 1 and 2 clinical trials. These trials involve various vaccines, with immune response being measured based on neutralizing antibody titers. Więcek et al [12] derived the efficacy of fractional doses against symptomatic infection by using its relationship with neutralizing antibody titers in standard doses [23]. However, how other clinical outcomes of COVID-19 (eg, asymptomatic infection) are related to measured immunity and how vaccine efficacies can be predicted by using dose fractions based on corresponding immunity levels remain unclear. In this study, we first investigated the relationship between neutralizing antibody titers and vaccine efficacies against SARS-CoV-2 infection. We then predicted vaccine efficacies for fractional doses by using real-world data obtained through a systematic review of neutralizing antibody levels induced by fractional doses of vaccines.

## Methods

### Sample and Data

To collect the neutralization data of COVID-19 vaccines that were reported in phase 1 and 2 clinical trial studies, we performed a systematic review of peer-reviewed studies in PubMed on March 15, 2022. We searched for studies in PubMed by using a combination of the following search terms, with no restriction on publication language: (1) *“COVID-19” OR “SARS-CoV-2” OR “2019-nCoV” OR “coronavirus”*; (2) “vaccin*”; (3) “fractiona*” OR “dose”; and (4) *“efficacy” OR “effectiveness” OR “neutralizing antibody” OR “neutralising antibody” OR “neutralization titer” OR “neutralization level” OR “antibody titer” OR “immune response” OR “immune protection” OR “immunogenicity” OR “reactogenicity” OR “safety” OR “adverse event” OR “adverse reaction” OR “adverse effect”*; the final search term included search terms 1 to 4, which were combined by using the Boolean operator *AND*. The searched studies were published between January 1, 2020, and March 15, 2022. The inclusion and exclusion criteria are summarized in Table 1. Studies were excluded if they were duplicate publications, preliminary animal studies, preprints, reviews, or commentaries. We reported studies in accordance with the PRISMA (Preferred Reporting Items for Systematic Reviews and Meta-Analyses) guidelines.

**Table 1.** Summarized inclusion and exclusion criteria. We performed a systematic review of peer-reviewed studies that reported on the neutralization of COVID-19 vaccines in phase 1 and 2 clinical trials and were published in PubMed between January 1, 2020, and March 15, 2022.

	Inclusion criteria	Exclusion criteria
Article type	Article types not listed in the exclusion criteria	Preliminary animal studies, preprints, reviews, and commentaries
Language	No restriction on publication language	—^a^
Article publication time	Between January 1, 2020, and March 15, 2022	Any time outside of that listed in the inclusion criteria

a Not applicable.

Information relevant to vaccines and participants was extracted from the selected studies, which included vaccine names, vaccine platforms, standard dosages (defined as the dosages determined for the approved vaccines or phase 3 trials), vaccination schedules, neutralization assays, neutralization measurement dates, the target viruses or pseudoviruses used to test the neutralizing antibody response, sample sizes and age groups of vaccinated participants, and sample sizes of convalescent cohorts (Multimedia Appendix 1). For each study, the mean neutralizing titers and their 95% CIs were extracted. Studies that did not report neutralizing titers of convalescent cohorts were excluded from further analysis. A random effects model or a fixed effects model was further used to perform a meta-analysis, according to the assessed heterogeneity between studies. In addition to the neutralization data from the phase 1 or 2 clinical trial studies, we collected the efficacy data of the vaccines from phase 3 studies, including the numbers of infections among vaccine groups and placebo groups. Data on 2 types of outcomes for vaccine efficacy were collected from the included phase 3 studies and assessed—symptomatic infection and asymptomatic infection outcomes (Multimedia Appendix 2).

### Study Selection

CL and YB assessed eligible studies, extracted relevant data, and conducted cross-checks. Conflicts regarding the study selection were resolved by another researcher (ZD). We excluded studies, based on the screening of titles and abstracts, if they (1) were reviews, commentaries, or preprints; (2) were not about SARS-CoV-2 vaccines; (3) were preliminary animal studies; (4) focused on full-dose vaccines; or (5) were not about neutralizing antibody response. We excluded studies, based on the full-text assessment, if they (1) lacked original neutralizing antibody data; (2) reported vaccines that were no longer being processed; or (3) reported vaccines for which standard dosages were not determined.

### Measures of Variables

We calculated the dose fraction for each sample as the fraction of the dose tested in the group divided by the corresponding standard dose. The mean neutralization level was defined as the ratio of the vaccine-induced neutralizing antibody level to the number of neutralizing antibodies in convalescent sera (ie, those measured in the same study). By doing so, we standardized the neutralizing antibody level and enabled the comparison of neutralization titers across studies that used different assays. The log_10_ transformation of the mean neutralization level was assumed to be normally distributed [23]. This log_10_-transformed mean neutralization level was the key variable in the models and data analyses reported in the *Results* section.

## Results

**Figure 1.**
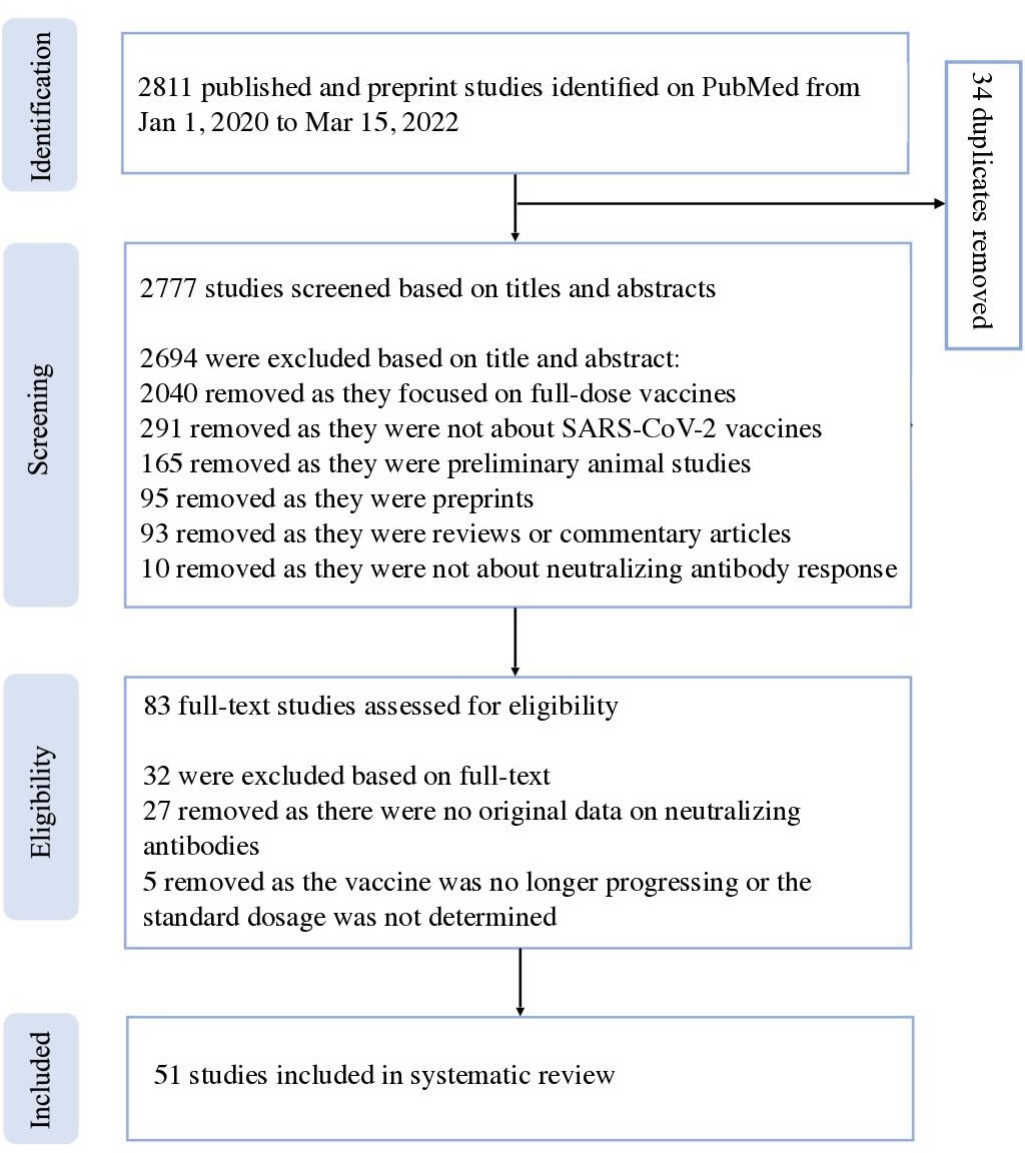
PRISMA (Preferred Reporting Items for Systematic Reviews and Meta-Analyses) flow diagram of our search for and selection of studies in the systematic review. We identified 2811 studies of COVID-19 vaccines, through the electronic search on PubMed, that were published between January 1, 2020, and March 15, 2022, and 51 studies were finally included in the systematic review.

We identified 2811 studies, through the electronic search on PubMed, that were published between January 1, 2020, and March 15, 2022. A total of 2777 studies were left after excluding duplicates. After 2694 studies were excluded based on the title and abstract screening, we retrieved 83 studies eligible for the full-text screening. After we excluded 32 studies based on the full-text screening, 51 studies met the inclusion criteria and were included in the systematic review (Figure 1, Multimedia Appendix 1, and Figure S1 in Multimedia Appendix 3).

**Figure 2.**
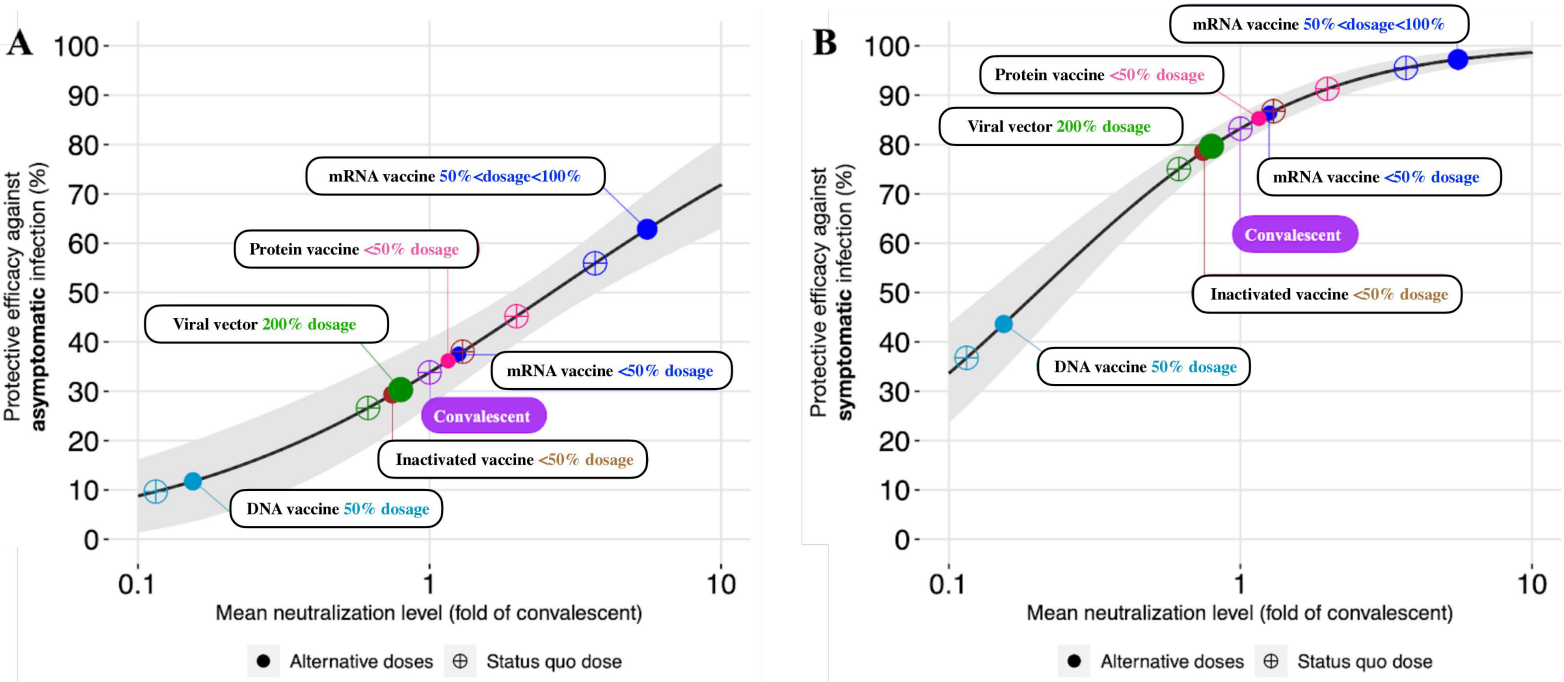
Estimating vaccine efficacy by using neutralizing antibody levels. Estimated vaccine efficacy against (A) asymptomatic infection and (B) symptomatic infection. The x-axis and y-axis denote the reported mean neutralization levels from phase 1 and 2 trials and the predicted protective efficacies for vaccines or the convalescent cohort, respectively. The gray solid line and shading indicate the best fit and the 95% CIs of the logistic model, respectively. Each dot indicates the predicted efficacy for vaccines (eg, mRNA, protein subunit, viral vector, inactivated, and DNA) or convalescent individuals.

Alongside the published studies included in the systematic review, we collected 13 records of the efficacy of 10 vaccines (eg, BNT162b2, ChAdOx1 nCoV-19, and CoronaVac) against asymptomatic and symptomatic infections for 11 COVID-19 variants (eg, Alpha, Beta, and Delta) from PubMed (Multimedia Appendix 2). Informed by the information on standard-dose vaccines, the nonlinear relationship between the standardized mean neutralization level and vaccine efficacies (which were stratified into 4 dosage groups) was fitted by using a logistic model (Figure S3 in Multimedia Appendix 3); the parameter estimates and root mean square deviations of the asymptomatic and symptomatic infection models are shown in Table 2. The slope parameter *k* for the asymptomatic infection model was estimated to be 1.84 and was smaller than that for the symptomatic infection model (3.10), reflecting that although vaccine efficacy increased with the mean neutralization level, the rate of this increase was slower for vaccine efficacy against asymptomatic infection than for vaccine efficacy against symptomatic infection (Figure 2). We estimated the mean neutralization level for 50% protection ($10^{n_{50}}$) against asymptomatic and symptomatic infections to be 262% (95% CI 190%‐361%) of the mean convalescent level and 20% (95% CI 14%‐28%) of the mean convalescent level, respectively. We predicted the vaccine efficacy of fractional doses against infection outcomes based on the established model (Figure 2). As the mean neutralization level increased from 0.1 to 10 folds of the convalescent level, the predicted vaccine efficacy against asymptomatic and symptomatic infections increased from 8.8% (95% CI 1.4%-16.1%) to 71.8% (95% CI 63%-80.7%) and from 33.6% (95% CI 23.6%-43.6%) to 98.6% (95% CI 97.6%-99.7%), respectively (Figure 2). mRNA vaccines provided the strongest protection, which decreased slowly for fractional dosing with dosages between 50% and 100% of the standard dose.

**Table 2.** Model parameter estimates for the logistic function of vaccine efficacy against asymptomatic and symptomatic infections. We fit the nonlinear relationship between the standardized mean neutralization level and vaccine efficacies (which were stratified into 4 dosage groups) by using a logistic model that was informed by the information on standard-dose COVID-19 vaccines (Figure S3 in Multimedia Appendix 3). The parameter estimates and root mean square deviations (RMSDs) of the asymptomatic and symptomatic infection models are shown in this table.

Parameter		Estimate (95% CI)^a^	RMSD, %
**Asymptomatic infection**			2.43
	Slope (*k*)	1.84 (1.15‐2.94)	
	50% protection ($10^{n_{50}}$)	2.62 (1.90‐3.61)^b^	
**Symptomatic infection**			0.16
	Slope (*k*)	3.10 (2.19‐4.38)	
	50% protection ($10^{n_{50}}$)	0.20 (0.14‐0.28)^b^	

a Parameter estimates and CIs for the logistic function (Equation 1 in Multimedia Appendix 3) describe the protective efficacy experienced by individuals with a given log_10_-transformed neutralization level.

b The 50% protective neutralization values reported here have been converted from the log_10_ scale back to the linear scale (as reported in the main text).

**Figure 3.**
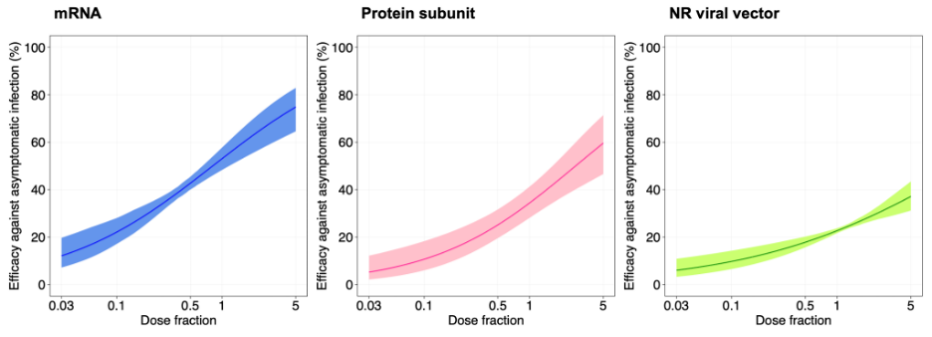
Estimating vaccine efficacy against asymptomatic infection by using neutralizing antibody levels of vaccines. The x-axis and y-axis denote the fraction of dose and the estimated protective efficacy for 3 types of vaccines (eg, mRNA, protein subunit, and NR viral vector), respectively. The solid line and shading indicate the best fit and the 95% CIs of the logistic model, respectively. NR: nonreplicating.

**Figure 4.**
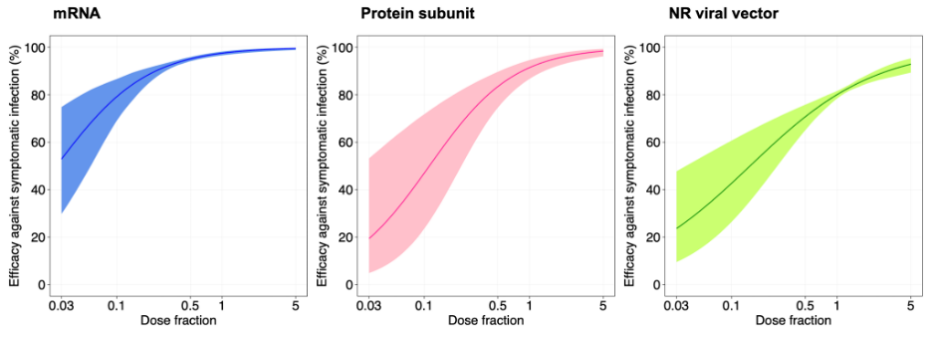
Estimating vaccine efficacy against symptomatic infection by using neutralizing antibody levels of vaccines. The x-axis and y-axis denote the fraction of dose and the estimated protective efficacy for 3 types of vaccines (eg, mRNA, protein subunit, and NR viral vector), respectively. The solid line and shading indicate the best fit and the 95% CIs of the logistic model, respectively. NR: nonreplicating.

The results of fitting the generalized additive model on dose fractions and mean neutralization levels are presented in Table S1 in Multimedia Appendix 3. The estimated coefficients of the log_10_ of the dose fractions were 0.746 (*P*=.02), 0.803 (*P*=.002), and 0.543 (*P*=.02) for mRNA, protein subunit, and nonreplicating viral vector vaccines, respectively. The fitted models showed that the efficacies of mRNA (*P*=.02) and protein subunit (*P*=.002) vaccines significantly increased as the dose fraction increased (Figure S4 in Multimedia Appendix 3). The predicted efficacies of dose fractions for different vaccine types are presented in Figures 2-4. For example, the mRNA vaccine efficacy against asymptomatic and symptomatic infections increased from 12.1% (95% CI 7.2%-19.7%) to 74.8% (95% CI 64.6%-82.9%) and from 52.9% (95% CI 29.8%-74.8%) to 99.5% (95% CI 98.9%-99.8%), respectively, for dose fractions ranging from 0.03 to 5. The protein subunit vaccines had efficacies of 5.2% (95% CI 2.1%-12.2%) to 59.6% (95% CI 46.5%-71.4%) against asymptomatic infection and 19.4% (95% CI 4.8%-53.2%) to 98.4% (95% CI 96.2%-99.3%) against symptomatic infection for dose fractions in the study range of 0.03 to 5. The nonreplicating viral vector vaccines had efficacies of 6% (95% CI 3.3%-10.9%) to 37.1% (95% CI 31.2%-43.4%) against asymptomatic infection and 23.6% (95% CI 9.5%-47.7%) to 92.9% (95% CI 89.3%-95.3%) against symptomatic infection for dose fractions in the study range of 0.03 to 5.

## Discussion

This review revealed the immunogenicity, efficacy, and safety of fractional doses as of December 2021, indicating that they would be safe and comparably effective to the standard doses. The included articles predominantly focused on 10 vaccines (eg, BNT162b2, ChAdOx1 nCoV-19, and CoronaVac) for 11 COVID-19 variants (eg, Alpha, Beta, and Delta). Informed by available data from published studies of immune responses for both standard doses and fractional doses, we predicted vaccine efficacies by using neutralizing antibody levels for both standard-dose vaccines and fractional-dose vaccines.

We investigated the relationship between neutralizing antibody titers and the vaccine efficacies against infection for standard doses and derived vaccine efficacies for fractional doses. This study contributes further evidence supporting the use of neutralization titers as significant predictors of vaccine efficacy against asymptomatic infections, symptomatic infections, and severe diseases, in line with previous studies [23,24]. We additionally explored the relationship between dose fractions and vaccine efficacy, using neutralization titers as intermediate variables and accounting for vaccine type.

There would be public health and economic advantages to using a dose-sparing strategy to increase vaccine supply and vaccination coverage around the world [12,20]. According to the World Health Organization, fractional dosing strategies have the potential to save lives, but only after a thorough review of immunogenicity data [25]. Using untested fractionated vaccine doses in a low-resource setting should also be considered, that is, in cases where higher-resource settings have access to the full vaccine doses and ethics and politics have been taken into account [20]. Further, fractional-dose vaccination could help to optimize the availability of vaccine doses during vaccine shortages, and even in cases where the vaccine supply is increasing, this strategy would still have a high value for vulnerable individuals, as lower doses may also be linked with fewer side effects [17-19].

The following five vaccine types were included in the final analysis: mRNA, protein subunit, nonreplicating viral vector, inactivated, and DNA vaccines. Other vaccine types and mix-and-match vaccine regimens have been used in the rollout of booster programs. For example, previous studies showed that the mix-and-match CoronaVac/BNT162b2 vaccination regimen was superior when compared to the CoronaVac/CoronaVac regimen in terms of immunogenicity [26]. As more studies are conducted and evidence accumulates, our model can be expanded to incorporate combinations of vaccines, thereby enhancing its robustness and utility. Given that the immunity and efficacy data we used were all from the general population, our results may not apply to specific groups, such as individuals who are immunocompromised or high-risk occupational populations, since neutralization levels and efficacies in these groups can be different [27,28].

Despite the vital role of a timely vaccination plan for reducing negative pandemic impacts in society [29,30], studies have suggested that manifold factors (eg, psychological, sociodemographic, cultural, institutional, environmental, and economic factors) in society that drive infections and numbers of deaths should also be considered and emphasized in plans for facing the next pandemic crisis [31-33].

Despite the robustness of our qualitative results, we identified certain limitations. First, our study does not explicitly include age groups, across which levels of vaccine efficacy and safety may differ [34]. Second, our study does not include the waning rate of vaccine-derived immunity for fractional-dose vaccination, which may vary with respect to dose size [12]. Third, the neutralizing antibody data were extracted for ancestral stains, but data on efficacy against asymptomatic infection were collected for different variants. Ideally, we should have used the neutralizing antibody and efficacy data of the same strains, but the neutralizing antibody data of the different variants were limited, and the neutralizing antibody levels of the different variants may have differed from those of the ancestral strains [35-37]. Hence, our prediction of efficacy against COVID-19 variants was based on neutralization levels specific to ancestral strains. Caution is advised when applying the model; however, it remains valid if neutralization levels of ancestral strains are tested and used as the predictor. Our findings provide a theoretical basis for the efficacy of fractional-dose vaccines, serving as reference evidence for implementing fractional dosing vaccine policies in areas with vaccine shortages to reduce disease burden. Effective governance would facilitate the implementation of fractional dosing strategies at the early phase of the next pandemic, thereby providing time for vaccine production to increase and meet supply and demand requirements [6].

To summarize, our results are consistent with studies on predicting immune protection against symptomatic SARS-CoV-2 infection [23], and we further projected vaccine protection against asymptomatic infection. We expect that fractional-dose vaccination could provide partial immunity against, for example, SARS-CoV-2 infection. This strategy would be cost-effective in curbing the COVID-19 outbreak, as it balances the lower efficacy of smaller doses with faster vaccination coverage and lower side effects, especially if global vaccine shortage issues persist.

## Supplementary material

10.2196/49812 Multimedia Appendix 1 Information extracted from the selected studies.

10.2196/49812 Multimedia Appendix 2 Symptomatic infection and asymptomatic infection outcomes.

10.2196/49812 Multimedia Appendix 3 Data analysis procedure and further results from our models.

10.2196/49812 Checklist 1 PRISMA (Preferred Reporting Items for Systematic Reviews and Meta-Analyses) checklist.

## Abbreviations

FDA: US Food and Drug Administration
mpox: monkeypox
PRISMA: Preferred Reporting Items for Systematic Reviews and Meta-Analyses
