# Supplementary material for: Predicting Efficacies of Fractional Doses of Vaccines by Using Neutralizing Antibody Levels: Systematic Review and Meta-Analysis
Source: JMIR Public Health Surveill. 2024 Jul 12;10:e49812. doi: 10.2196/49812 (PMC11259582; doi:10.2196/49812)
Supplement: Multimedia Appendix 3 [file publichealth-v10-e49812-s003.docx]

# **Supplement**

Models and Data analysis procedure

Informed by the mean neutralization levels induced by vaccines of standard dose and their efficacies data from eligible studies, we fitted the logistic model to study the relationship between neutralization and protection against infection outcomes ([
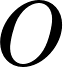
](https://www.codecogs.com/eqnedit.php?latex=o#0), i.e., asymptomatic or symptomatic infection), following Ref. [[23]](https://paperpile.com/c/hnsVdC/KEs38) ([Figure S4](https://docs.google.com/document/d/1RZlQaVxUempZKC7A-ll-i4W0NMM4nAMlQppZ6XC-5es/edit#sif_fit)):

[
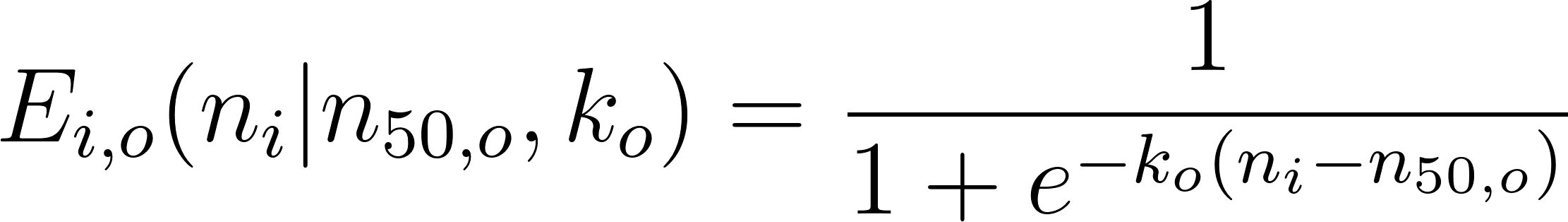
](https://www.codecogs.com/eqnedit.php?latex=E_%7Bi%2C%20o%7D(n_i%7Cn_%7B50%2C%20o%7D%2C%20k_o)%20%3D%20%5Cfrac%7B1%7D%7B1%2Be%5E%7B-k_o(n_i-n_%7B50%2Co%7D)%7D#0), (1)

where [
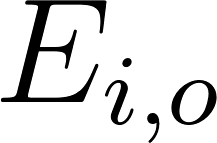
](https://www.codecogs.com/eqnedit.php?latex=E_%7Bi%2Co%7D#0) is the protective efficacy of an individual [
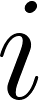
](https://www.codecogs.com/eqnedit.php?latex=i#0) against infection outcome [
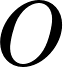
](https://www.codecogs.com/eqnedit.php?latex=o#0) given the neutralization level [
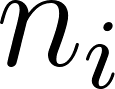
](https://www.codecogs.com/eqnedit.php?latex=n_i#0), and [
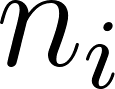
](https://www.codecogs.com/eqnedit.php?latex=n_i#0) is the log10 transform of the mean neutralization level. The paramater [
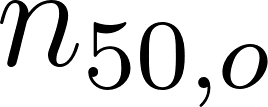
](https://www.codecogs.com/eqnedit.php?latex=n_%7B50%2C%20o%7D#0) denotes the log10-transformed mean neutralization level at which an individual will have 50% efficacy against infection outcome [
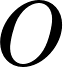
](https://www.codecogs.com/eqnedit.php?latex=o#0) (that is, half the chance of being infected compared with an unvaccinated person), and [
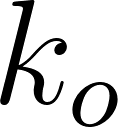
](https://www.codecogs.com/eqnedit.php?latex=k_o#0) is the steepness of this model. We assumed [
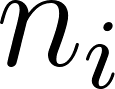
](https://www.codecogs.com/eqnedit.php?latex=n_i#0) follows a normal distribution with mean [
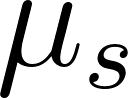
](https://www.codecogs.com/eqnedit.php?latex=%5Cmu_s#0) and standard deviation [
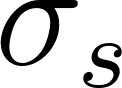
](https://latex-staging.easygenerator.com/eqneditor/editor.php?latex=%5Csigma_s#0) in study [
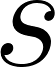
](https://www.codecogs.com/eqnedit.php?latex=s#0). The proportion of the vaccinated population that will be protected is given by:

[
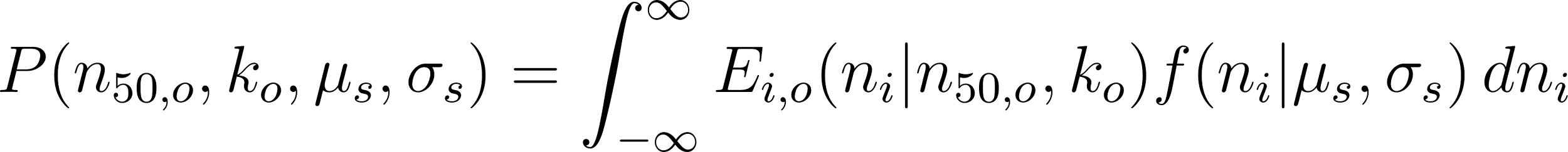
](https://www.codecogs.com/eqnedit.php?latex=P(n_%7B50%2Co%7D%2Ck_o%2C%5Cmu_s%2C%5Csigma_s)%3D%5Cint_%7B-%5Cinfty%7D%5E%7B%5Cinfty%7DE_%7Bi%2Co%7D(n_i%7Cn_%7B50%2Co%7D%2Ck_o)f(n_i%7C%5Cmu_s%2C%5Csigma_s)%5C%2Cdn_i%5C#0), (2)

where [
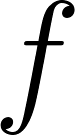
](https://www.codecogs.com/eqnedit.php?latex=f#0) is the normal probability density function of [
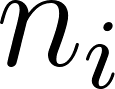
](https://www.codecogs.com/eqnedit.php?latex=n_i#0), and [
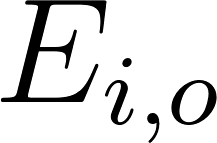
](https://www.codecogs.com/eqnedit.php?latex=E_%7Bi%2Co%7D#0) is the logistic function in equation (1). The mean parameter [
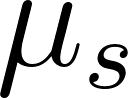
](https://www.codecogs.com/eqnedit.php?latex=%5Cmu_s#0) was estimated by log10 transform of the mean neutralization level of each study, and [
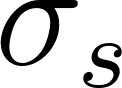
](https://latex-staging.easygenerator.com/eqneditor/editor.php?latex=%5Csigma_s#0) was estimated as [
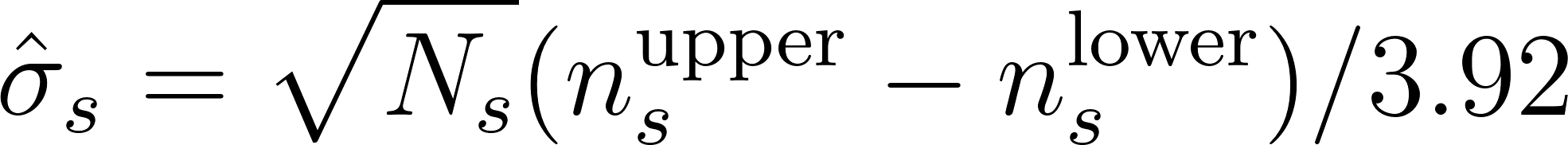
](https://www.codecogs.com/eqnedit.php?latex=%5Chat%7B%5Csigma%7D_s%3D%5Csqrt%7BN_s%7D(n_s%5E%7B%5Ctext%7Bupper%7D%7D-n_s%5E%7B%5Ctext%7Blower%7D%7D)%2F3.92#0), where [
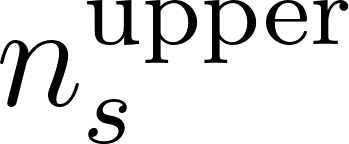
](https://www.codecogs.com/eqnedit.php?latex=n_s%5E%5Ctext%7Bupper%7D#0) and [
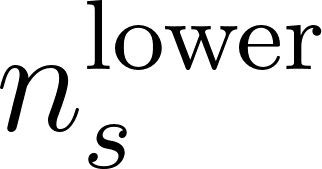
](https://www.codecogs.com/eqnedit.php?latex=n_s%5E%5Ctext%7Blower%7D#0) denote the upper and lower bound of the 95% CI of [
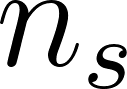
](https://www.codecogs.com/eqnedit.php?latex=n_s#0), respectively, and [
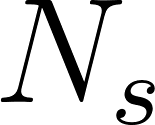
](https://www.codecogs.com/eqnedit.php?latex=N_s#0) is the sample size of the phase 3 study [
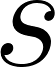
](https://www.codecogs.com/eqnedit.php?latex=s#0).

From the included phase 3 clinical trials, we extracted the number of participants in placebo group [
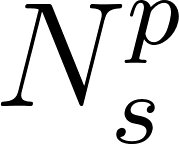
](https://www.codecogs.com/eqnedit.php?latex=N_s%5Ep#0), the number of participants in vaccine group [
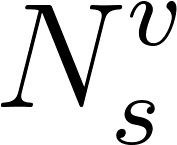
](https://www.codecogs.com/eqnedit.php?latex=N_s%5Ev#0), the number of infected participants in placebo group [
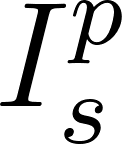
](https://latex-staging.easygenerator.com/eqneditor/editor.php?latex=I_s%5Ep#0), the number of infected participants in vaccine group [
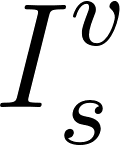
](https://www.codecogs.com/eqnedit.php?latex=I_s%5Ev#0), and obtained the likelihood of the observed infections of outcome [
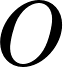
](https://www.codecogs.com/eqnedit.php?latex=o#0) in study [
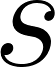
](https://www.codecogs.com/eqnedit.php?latex=s#0):

[
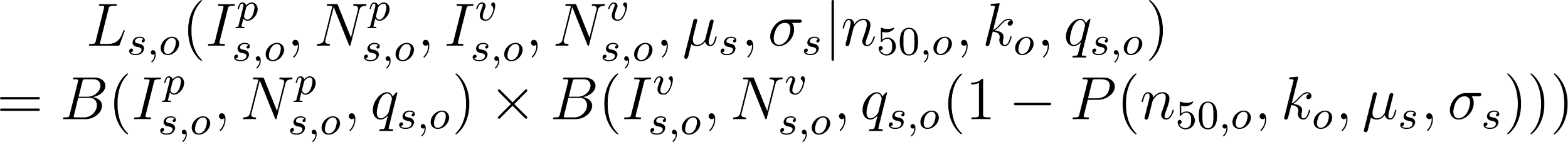
](https://www.codecogs.com/eqnedit.php?latex=L_%7Bs%2Co%7D(I_%7Bs%2Co%7D%5Ep%2C%20N_%7Bs%2Co%7D%5Ep%2C%20I_%7Bs%2Co%7D%5Ev%2C%20N_%7Bs%2Co%7D%5Ev%2C%20%5Cmu_s%2C%20%5Csigma_s%20%7C%20n_%7B50%2Co%7D%2Ck_o%2Cq_%7Bs%2Co%7D)%20%5C%5C%5C%5C%20%3D%20B(I_%7Bs%2Co%7D%5Ep%2C%20N_%7Bs%2Co%7D%5Ep%2C%20q_%7Bs%2Co%7D)%20%20%5Ctimes%20%20B(I_%7Bs%2Co%7D%5Ev%2C%20N_%7Bs%2Co%7D%5Ev%2C%20q_%7Bs%2Co%7D(1-P(n_%7B50%2Co%7D%2Ck_o%2C%5Cmu_s%2C%5Csigma_s)))#0), (3)

where [
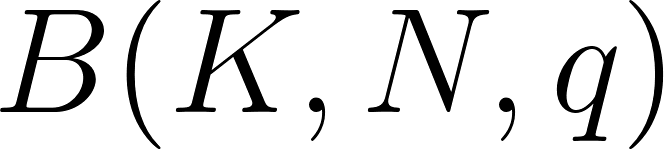
](https://www.codecogs.com/eqnedit.php?latex=B(K%2C%20N%2C%20q)#0) denotes the binomial probability mass function of the probability of getting [
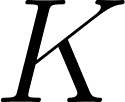
](https://www.codecogs.com/eqnedit.php?latex=K#0) events from a sample size of [
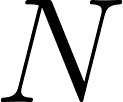
](https://www.codecogs.com/eqnedit.php?latex=N#0) with a success probability [
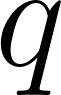
](https://www.codecogs.com/eqnedit.php?latex=q#0). [
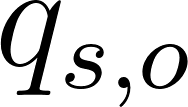
](https://www.codecogs.com/eqnedit.php?latex=q_%7Bs%2Co%7D#0) is the probability of an unvaccinated individual to develop infection outcome [
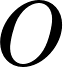
](https://www.codecogs.com/eqnedit.php?latex=o#0) in study [
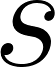
](https://www.codecogs.com/eqnedit.php?latex=s#0), [
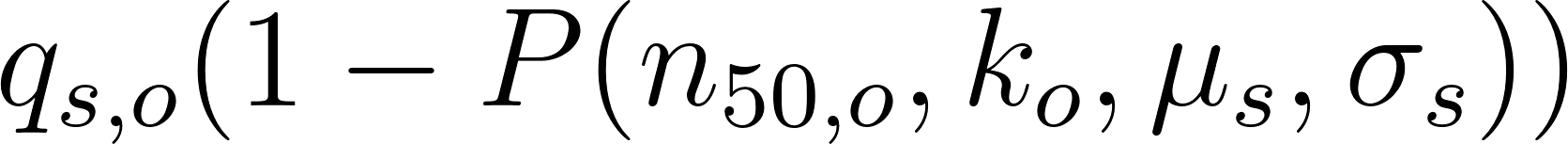
](https://www.codecogs.com/eqnedit.php?latex=q_%7Bs%2Co%7D(1-P(n_%7B50%2Co%7D%2Ck_o%2C%5Cmu_s%2C%5Csigma_s))#0) is that probability in the vaccine group accordingly. The likelihood of observing infections of outcome [
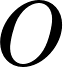
](https://www.codecogs.com/eqnedit.php?latex=o#0) for all studies is the product of the likelihood for each study:

[
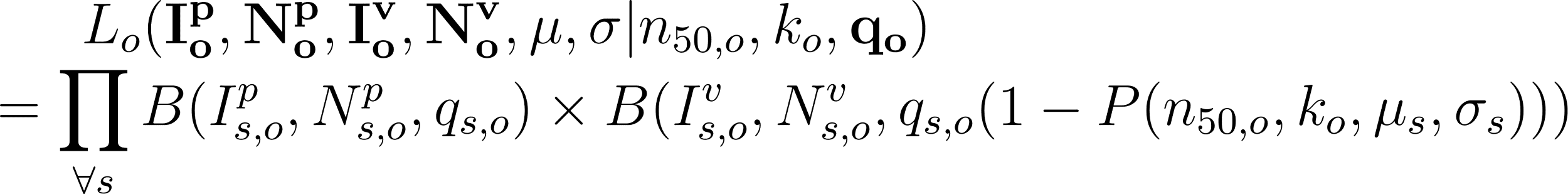
](https://www.codecogs.com/eqnedit.php?latex=L_o(%5Cmathbf%7BI_o%5Ep%7D%2C%5Cmathbf%7BN_o%5Ep%7D%2C%20%5Cmathbf%7BI_o%5Ev%7D%2C%20%5Cmathbf%7BN_o%5Ev%7D%2C%20%5Cmathbf%7B%5Cmu%7D%2C%20%5Cmathbf%7B%5Csigma%7D%20%7C%20n_%7B50%2Co%7D%2C%20k_o%2C%20%5Cmathbf%7Bq_o%7D)%5C%5C%5C%5C%20%3D%20%5Cprod_%7B%5Cforall%20s%7DB(I_%7Bs%2Co%7D%5Ep%2C%20N_%7Bs%2Co%7D%5Ep%2C%20q_%7Bs%2Co%7D)%20%20%5Ctimes%20%20B(I_%7Bs%2Co%7D%5Ev%2C%20N_%7Bs%2Co%7D%5Ev%2C%20q_%7Bs%2Co%7D(1-P(n_%7B50%2Co%7D%2Ck_o%2C%5Cmu_s%2C%5Csigma_s)))#0). (4)

The maximum likelihood estimates of [
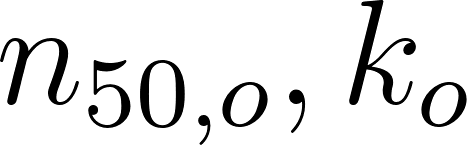
](https://www.codecogs.com/eqnedit.php?latex=n_%7B50%2Co%7D%2C%20k_o#0) and [
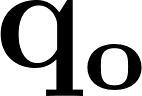
](https://www.codecogs.com/eqnedit.php?latex=%5Cmathbf%7Bq_o%7D#0) were obtained using nlm function in R (programming language) by minimizing -log (Lo). The standard error of the estimates was computed using the output Hessian matrix H as se = [
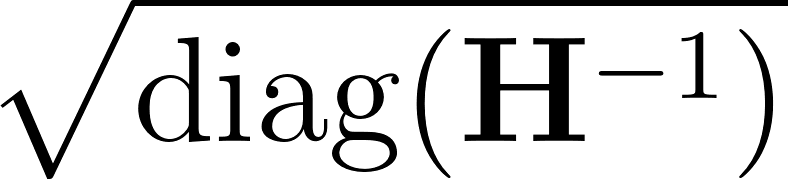
](https://www.codecogs.com/eqnedit.php?latex=%5Csqrt%7B%5Ctext%7Bdiag%7D(%5Cmathbf%7BH%7D%5E%7B-1%7D)%7D#0), and the 95% CIs were calculated as $\pm$1.96 $\times$ se of the estimates.

Using the fitted logistic model, we projected the vaccine efficacy against asymptomatic and symptomatic infection over fractional dosing for each vaccine type. We categorized different doses into four groups, i.e., dose <50%, dose = 50%, 50% < dose < 100%, and dose = 200%. For each vaccine type included in the final analyses, namely, mRNA, protein subunit, non-replicating viral vector, inactivated, and DNA vaccine, we pooled the log10-transformed mean neutralization levels by dose group and predicted the efficacy of each dose group using the pooled mean. To perform pool analysis, for dose group [
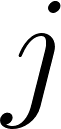
](https://www.codecogs.com/eqnedit.php?latex=j#0) in study [
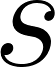
](https://www.codecogs.com/eqnedit.php?latex=s#0), we used bootstrap method to estimate the 95% confidence intervals of the mean [
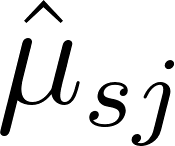
](https://www.codecogs.com/eqnedit.php?latex=%5Chat%7B%5Cmu%7D_%7Bsj%7D#0) and standard deviation [
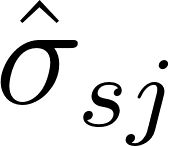
](https://www.codecogs.com/eqnedit.php?latex=%5Chat%7B%5Csigma%7D_%7Bsj%7D#0) of the log10-transformed mean neutralization levels. Based on the normal distribution with estimated mean [
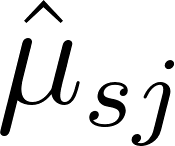
](https://www.codecogs.com/eqnedit.php?latex=%5Chat%7B%5Cmu%7D_%7Bsj%7D#0) and standard deviation [
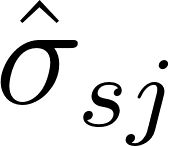
](https://www.codecogs.com/eqnedit.php?latex=%5Chat%7B%5Csigma%7D_%7Bsj%7D#0) from 2000 bootstrap samples, we obtained the 95% confidence intervals as [
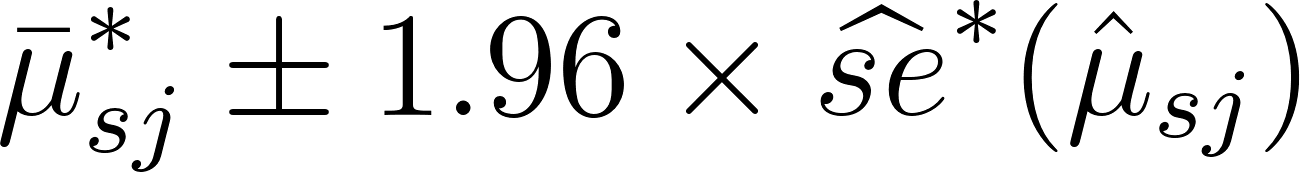
](https://www.codecogs.com/eqnedit.php?latex=%5Cbar%7B%5Cmu%7D%5E*_%7Bsj%7D%5Cpm1.96%5Ctimes%20%5Cwidehat%7Bse%7D%5E*(%5Chat%7B%5Cmu%7D_%7Bsj%7D)#0) and [
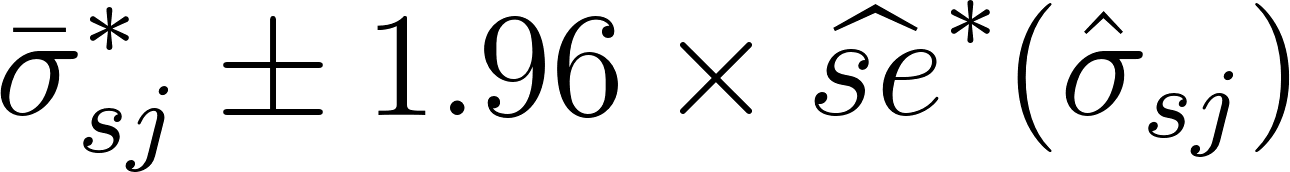
](https://www.codecogs.com/eqnedit.php?latex=%5Cbar%7B%5Csigma%7D%5E*_%7Bsj%7D%5Cpm1.96%5Ctimes%20%5Cwidehat%7Bse%7D%5E*(%5Chat%7B%5Csigma%7D_%7Bsj%7D)#0), where [
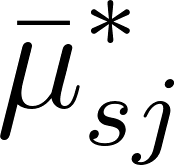
](https://www.codecogs.com/eqnedit.php?latex=%5Cbar%7B%5Cmu%7D%5E*_%7Bsj%7D#0) and [
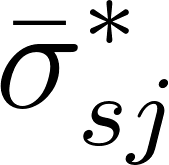
](https://www.codecogs.com/eqnedit.php?latex=%5Cbar%7B%5Csigma%7D%5E*_%7Bsj%7D#0) are the mean of bootstrap replicates for [
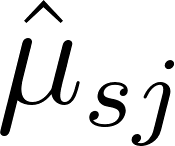
](https://www.codecogs.com/eqnedit.php?latex=%5Chat%7B%5Cmu%7D_%7Bsj%7D#0) and [
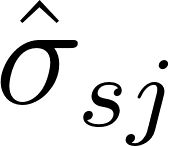
](https://www.codecogs.com/eqnedit.php?latex=%5Chat%7B%5Csigma%7D_%7Bsj%7D#0), respectively. By substituting the pooled log10-transformed mean neutralization level for [
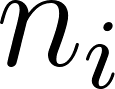
](https://www.codecogs.com/eqnedit.php?latex=n_i#0) into equation (1), we obtained the predicted vaccine efficacy for each fractional dosing (Figure 1).

Our secondary analysis was to illustrate the dose-efficacy relationship for different vaccines, we first quantified the relationship between dose fraction and mean neutralization levels using a generalized additive model such that:

[
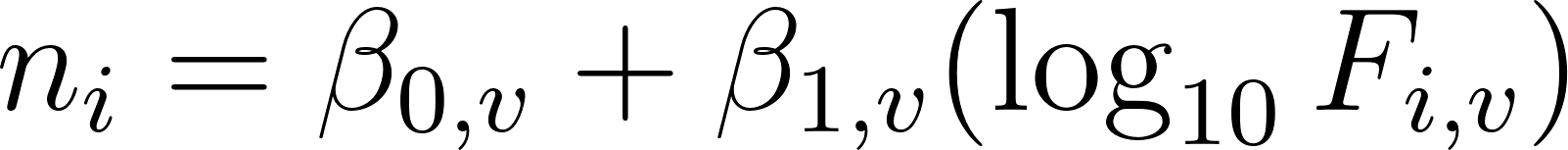
](https://www.codecogs.com/eqnedit.php?latex=n_i%20%3D%20%5Cbeta_%7B0%2Cv%7D%20%2B%20%5Cbeta_%7B1%2Cv%7D(%5Clog_%7B10%7DF_%7Bi%2Cv%7D)%20#0), (5)

where [
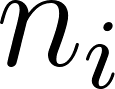
](https://www.codecogs.com/eqnedit.php?latex=n_i#0) is the log10-transformed mean neutralization level of individual [
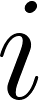
](https://www.codecogs.com/eqnedit.php?latex=i#0), [
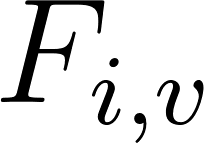
](https://www.codecogs.com/eqnedit.php?latex=F_%7Bi%2Cv%7D#0) is the dose fraction of vaccine type [
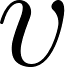
](https://www.codecogs.com/eqnedit.php?latex=v#0) (Figure 2). A non-parametric bootstrap method was conducted to obtain the estimated standard error of [
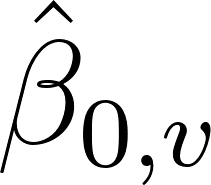
](https://www.codecogs.com/eqnedit.php?latex=%5Chat%7B%5Cbeta%7D_%7B0%2Cv%7D#0) and [
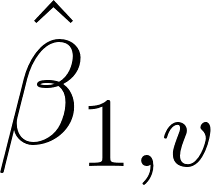
](https://www.codecogs.com/eqnedit.php?latex=%5Chat%7B%5Cbeta%7D_%7B1%2Cv%7D#0) based on 2000 replicates. Combining Eq. (5) with equation (1), the dose-efficacy relationship was derived (Figure 2-3). Analyses were conducted in R version 4.1.1.

**
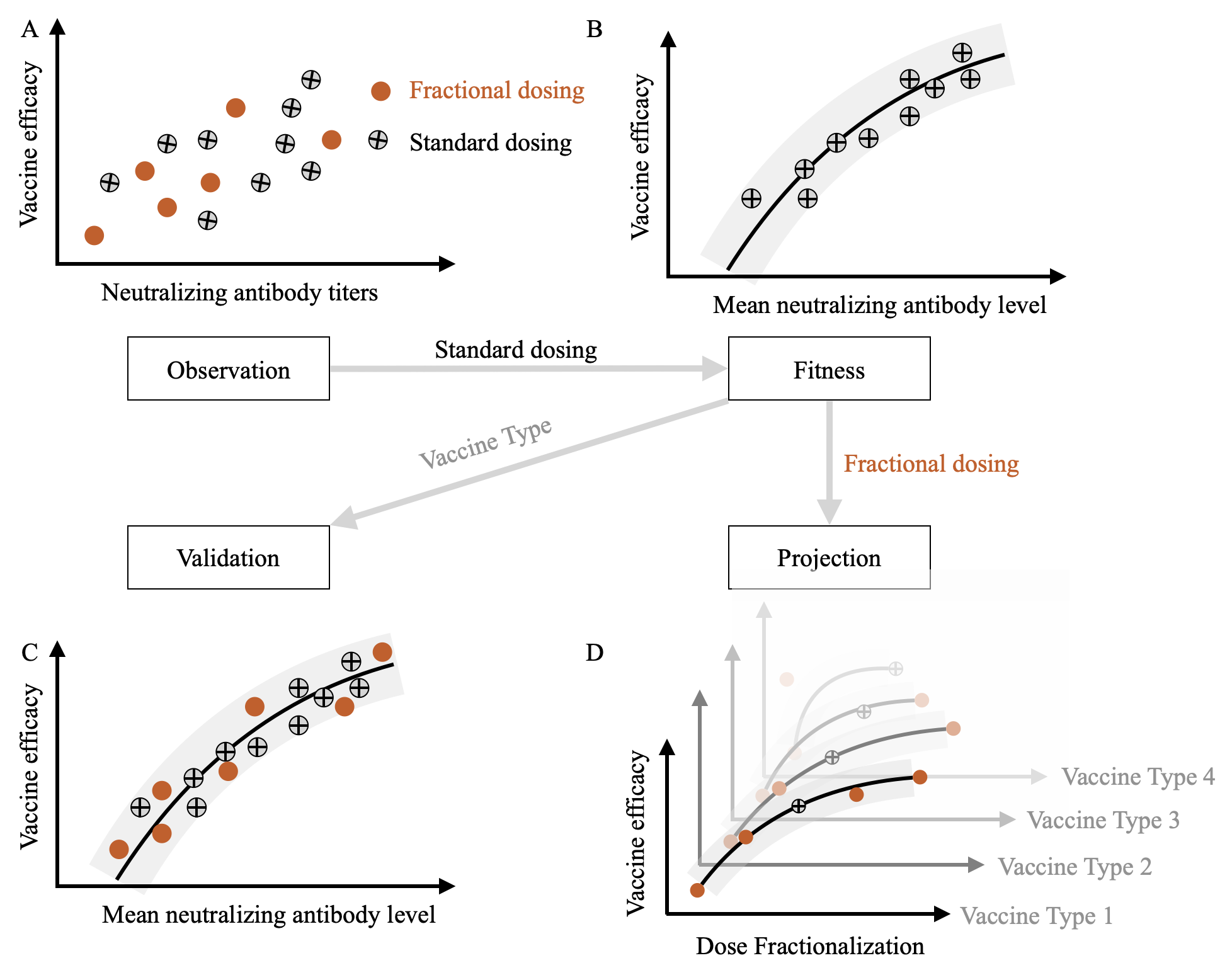
**

[**Figure S1**](https://docs.google.com/document/d/1igVguV_fJz67t-Wk6nz-oTk3woFJoYnrlGDOu3D3Pv4/edit#sifig_scheme)**. Schematic illustration of the fitting and projecting methods for vaccine efficacies.** First, we collect clinical trial data from published studies ([**Figure S2**](https://docs.google.com/document/d/1Eo0FOf6Fm_O6JtM-iJbf_Sza1LaeoS0OIr12c6XWNAs/edit#sif_PRISMA)) for both vaccine efficacy and neutralizing antibody (NAb) titers across six types of vaccines ([**Figure S3**](https://docs.google.com/document/d/1Eo0FOf6Fm_O6JtM-iJbf_Sza1LaeoS0OIr12c6XWNAs/edit#sif_temporal)). Second, we estimate the relationship between the mean neutralizing antibody level and each study vaccine efficacy using the logistic model ([**Figure S4**](https://docs.google.com/document/d/1Eo0FOf6Fm_O6JtM-iJbf_Sza1LaeoS0OIr12c6XWNAs/edit#sif_fit)). We then validate the relationship by fractional dosing ([**Figure 1**](https://docs.google.com/document/d/1Eo0FOf6Fm_O6JtM-iJbf_Sza1LaeoS0OIr12c6XWNAs/edit#mfi_1)) and project the vaccine efficacy for each type of vaccine along various dose fractionalization.

**
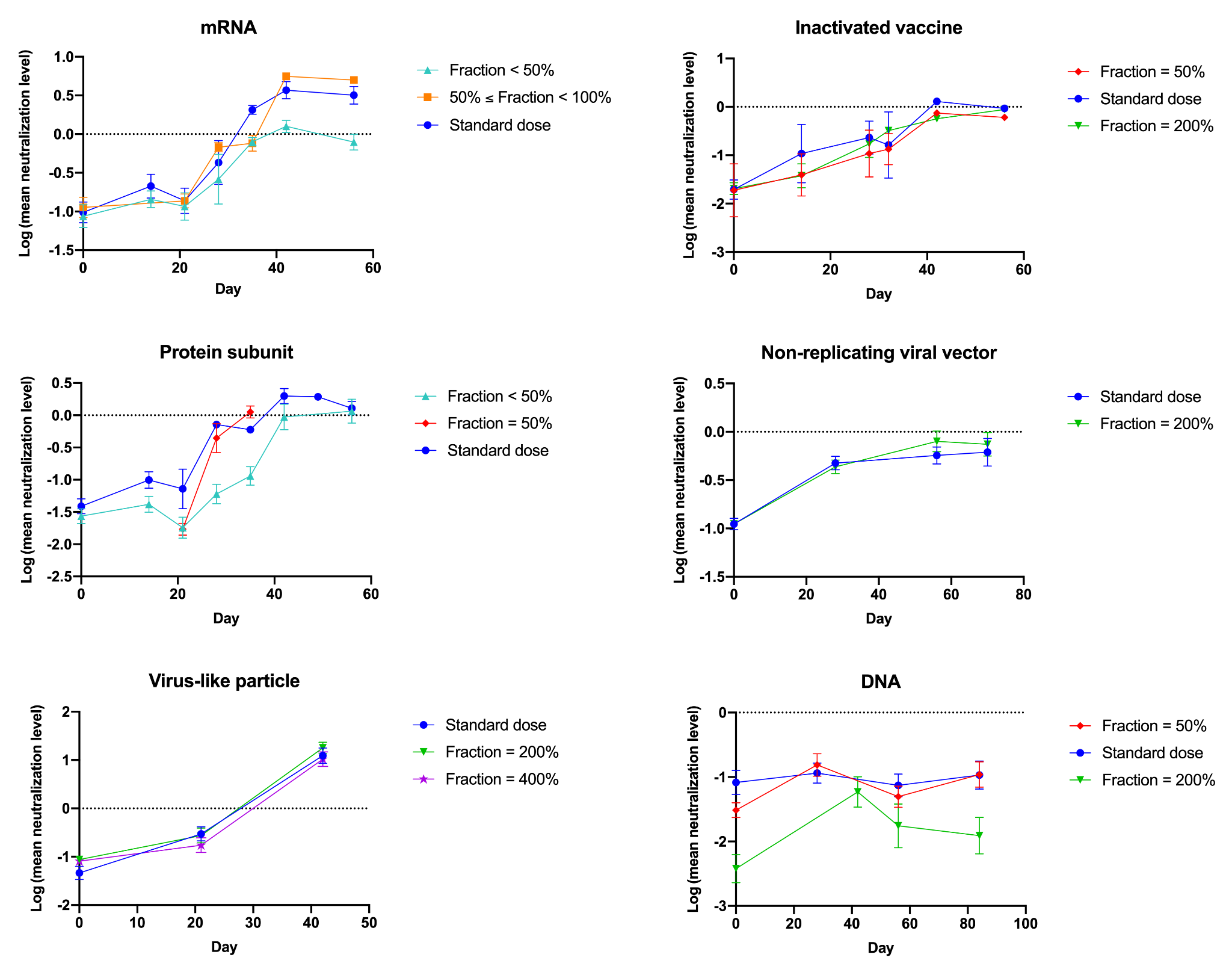
**

[**Figure S2**](https://docs.google.com/document/d/1igVguV_fJz67t-Wk6nz-oTk3woFJoYnrlGDOu3D3Pv4/edit#sifig_temporal)**. Neutralizing antibody levels over days of vaccines.** We collect the NAb dynamics of six types of vaccines. From surveyed studies, we collect values of mean neutralization level in a logarithmic scale for each of six vaccine types. For each type, we cluster vaccines according to their fractionalized dosage.

**
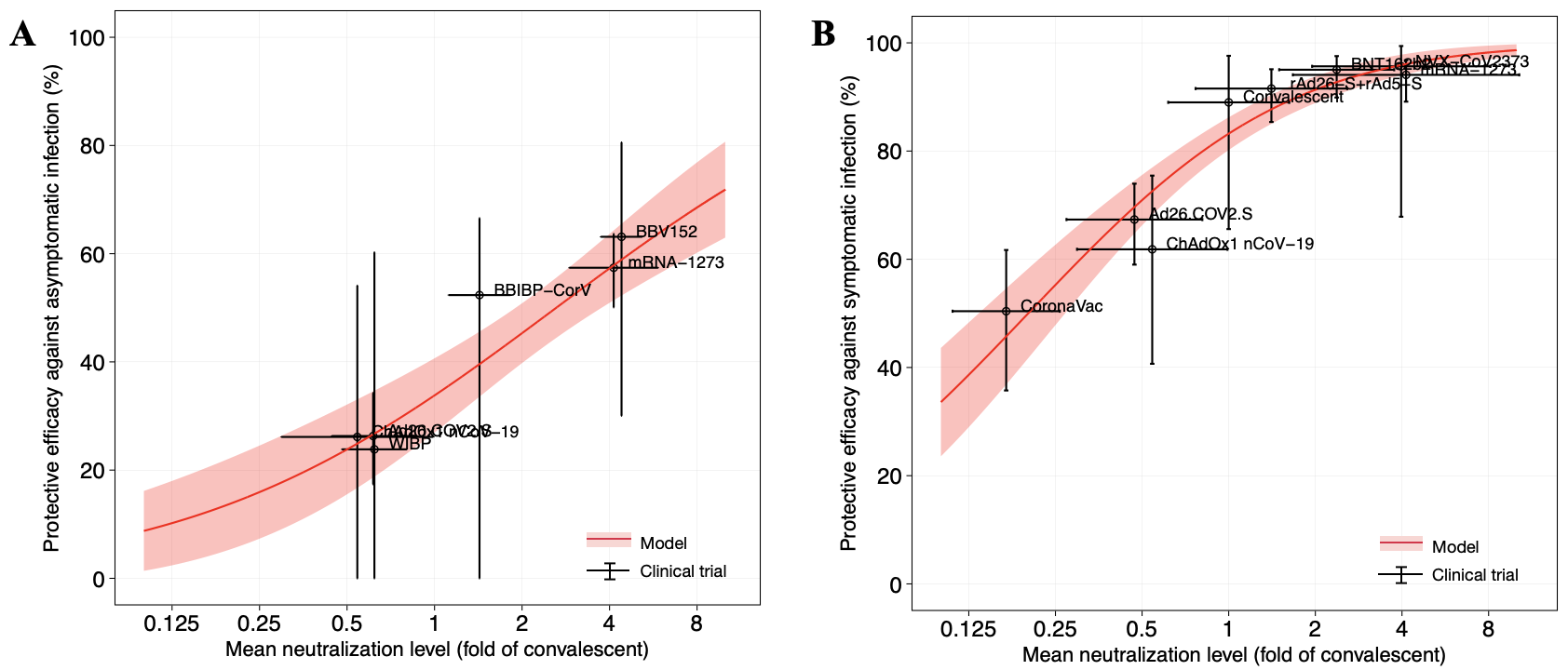
**

[**Figure S3**](https://docs.google.com/document/d/1igVguV_fJz67t-Wk6nz-oTk3woFJoYnrlGDOu3D3Pv4/edit#sifig_fit)**. Model fit of vaccine efficacies using neutralizing antibody levels.** Estimated vaccine efficacy against (**A**) asymptomatic infection and (B) symptomatic infection. The red solid line and shading indicate the best fit and the 95% confidence interval of the logistic model. Each dot indicates values from the reported mean neutralization level from phase 1 and 2 trials and the protective efficacy from phase 3 trials of standard-dose vaccines (e.g., mRNA, Protein subunit, viral vector, inactivated, and DNA).


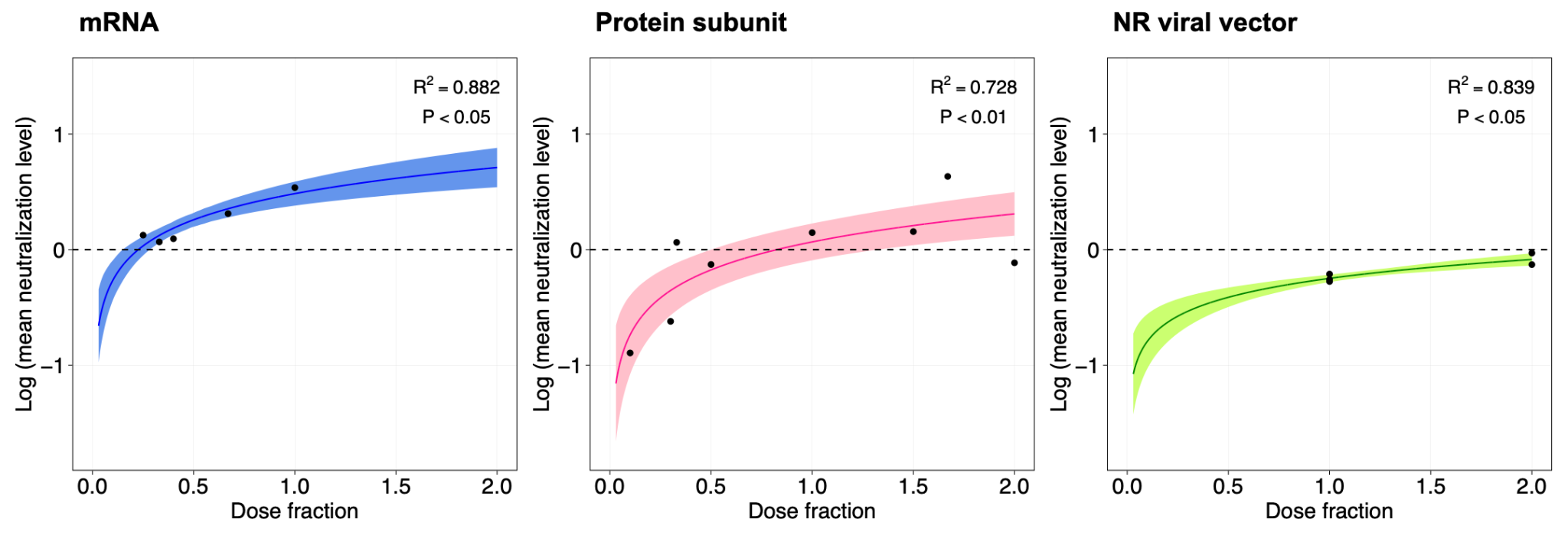


[**Figure S4**](https://docs.google.com/document/d/1igVguV_fJz67t-Wk6nz-oTk3woFJoYnrlGDOu3D3Pv4/edit#sifig_2)**. Model fit of neutralizing antibody levels using dose fraction.** The x-axis and y-axis denote the dose fraction and the estimated log_10_-transformed neutralizing antibody level for three types of vaccines (e.g., mRNA, Protein subunit, and Non-replicating viral vector), respectively. The solid line and shading indicate the best fit and the 95% confidence interval of the logistic model.

**Table S1**. Model parameter estimates for the generalized additive model on dose fraction and mean neutralization level

| **Coefficient** | **Estimate** | ***p*-Value** | **R^2^** |
| --- | --- | --- | --- |
| **mRNA** | | | |
| Intercept | 0.482 | 0.003 | 0.882 |
| Log_10_ of dose fraction | 0.746 | 0.015 |  |
| **Protein subunit** | | | |
| Intercept | 0.066 | 0.377 | 0.728 |
| Log_10_ of dose fraction | 0.803 | 0.002 |  |
| **NR viral vector** | | | |
| Intercept | -0.248 | 0.0007 | 0.839 |
| Log_10_ of dose fraction | 0.543 | 0.017 |  |
